# Transient Intervals of Hyper-Gravity Enhance Endothelial Barrier Integrity: Impact of Mechanical and Gravitational Forces Measured Electrically

**DOI:** 10.1371/journal.pone.0144269

**Published:** 2015-12-04

**Authors:** Robert Szulcek, Jan van Bezu, Johannes Boonstra, Jack J. W. A. van Loon, Geerten P. van Nieuw Amerongen

**Affiliations:** 1 Department of Physiology, Institute for Cardiovascular Research (ICaR-VU), VU University Medical Center, Amsterdam, The Netherlands; 2 Department of Pulmonology, Institute for Cardiovascular Research (ICaR-VU), VU University Medical Center, Amsterdam, The Netherlands; 3 Deptartment of Cellular Architecture and Dynamics, Science Faculty, Utrecht University, Utrecht, The Netherlands; 4 Dutch Experiment Support Center (DESC), ESTEC, TEC-MMG-Lab, European Space Agency (ESA), Noordwijk, The Netherlands; 5 Department of Oral and Maxillofacial Surgery/Oral Pathology, VU University Medical Center, Amsterdam, The Netherlands; 6 Department of Oral Cell Biology, Academic Centre for Dentistry (ACTA), Amsterdam, The Netherlands; University of South Florida, UNITED STATES

## Abstract

**Background:**

Endothelial cells (EC) guard vascular functions by forming a dynamic barrier throughout the vascular system that sensitively adapts to ‘classical’ biomechanical forces, such as fluid shear stress and hydrostatic pressure. Alterations in gravitational forces might similarly affect EC integrity, but remain insufficiently studied.

**Methods:**

In an unique approach, we utilized Electric Cell-substrate Impedance Sensing (ECIS) in the gravity-simulators at the European Space Agency (ESA) to study dynamic responses of human EC to simulated micro- and hyper-gravity as well as to classical forces.

**Results:**

Short intervals of micro- or hyper-gravity evoked distinct endothelial responses. Stimulated micro-gravity led to decreased endothelial barrier integrity, whereas hyper-gravity caused sustained barrier enhancement by rapid improvement of cell-cell integrity, evidenced by a significant junctional accumulation of VE-cadherin (p = 0.011), significant enforcement of peripheral F-actin (p = 0.008) and accompanied by a slower enhancement of cell-matrix interactions. The hyper-gravity triggered EC responses were force dependent and nitric-oxide (NO) mediated showing a maximal resistance increase of 29.2±4.8 ohms at 2g and 60.9±6.2 ohms at 4g vs. baseline values that was significantly suppressed by NO blockage (p = 0.011).

**Conclusion:**

In conclusion, short-term application of hyper-gravity caused a sustained improvement of endothelial barrier integrity, whereas simulated micro-gravity weakened the endothelium. In clear contrast, classical forces of shear stress and hydrostatic pressure induced either short-lived or no changes to the EC barrier. Here, ECIS has proven a powerful tool to characterize subtle and distinct EC gravity-responses due to its high temporal resolution, wherefore ECIS has a great potential for the study of gravity-responses such as in real space flights providing quantitative assessment of a variety of cell biological characteristics of any adherent growing cell type in an automated and continuous fashion.

## Introduction

Endothelial cells (EC) control vascular permeability by providing a dynamic barrier between blood and underlying tissue to regulate vascular functions, such as tissue perfusion and fluid homeostasis [1,2]. As for their location within the human body, EC are constantly exposed to fluid shear stress, cyclic stretch and hydrostatic pressure referred to as ‘classical’ biomechanical forces, whereby EC possess intrinsic capabilities to sense mechanical stimulations and dynamically adapt their morphology and function [3]. Loss of cell-cell junctional integrity caused by supra-physiological levels of classical forces leading to endothelial damage, hyper-permeability and vascular remodeling is associated with many pathological disorders [4,2] including pulmonary hypertension, edema, septic shock and atherosclerosis [5–7]. In addition to classical forces, EC are also exposed to brief alterations in gravity (g-forces) in our daily life, for example when we drive a car, conduct sports, use an elevator or travel by plane. Yet, the impact of short alterations in gravity on endothelial barrier integrity are unknown and gravity sensors are undefined [8–11].

Before the first space flights, g-forces were believed to have little to no effects on eukaryotic cells [12,13], but have now been proven to profoundly affect the cardiovascular system and cellular functions similarly to age related diseases [14]. Extensive periods of weightlessness showed detrimental effects on the human system defined as ‘cardiovascular deconditioning’ [15], but until now no targeted treatments are available to prevent the vascular impairments under real weightlessness as the underlying causes remain elusive [16]. In cultured cells, micro- and hyper-gravity are known to caused mainly opposite effects that are reversible at normal g-levels [17,11,18]. As such hyper-gravity induced enforcement of the cytoskeleton and cell migration [17,19], whereas micro-gravity led to loss of cytoskeletal integrity by dissociation of actin and tubulin bundles [20]. In addition, micro-gravity decreased wound healing capabilities in cells and small animal models and hampered responses to vasoactive stimulation [21,22]. Among others, these observations led to the assumption that the decrease in blood pressure and plasma volume during cardiovascular deconditioning might be caused by a loss of endothelial barrier integrity and increased vascular permeability in astronauts [23], which could be counter acted by the application of hyper-gravity. However, effects of altered gravity on the endothelium remain controversial and endothelial barrier integrity has never been quantified.

The lack of conclusive data describing the effects of g-forces on EC barrier function is due to rare test facilities, non-standardized experimental conditions and expensive space flights that limit experimental output to small sample numbers and single end-point measurements, which are insufficient to characterize the dynamic behavior of the endothelium [17]. More importantly, most standard assays for the characterization of endothelial barrier function (like macro-molecule passage) are incompatible with space laboratory conditions and ground-based gravity-simulators, wherefore an urgent demand for innovative measurements to characterize cellular behavior exists. To overcome these limitations, we utilized Electric Cell-substrate Impedance Sensing (ECIS) in the Large Diameter Centrifuge (LDC) and the Random Positioning Machine (RPM) at the European Space Research and Technology Center (ESA-ESTEC) aiming to study EC gravity-responses under simulated conditions of altered gravity and test applicability of the ECIS technology for this type of studies and real space flights. ECIS is an easy to use, non-invasive electrical measurement system to quantify several cell biological processes, such as barrier integrity, cell adhesions, motility and migration as well as responses to drugs and toxins in a single, automated, continuous measurement with a high temporal resolution [24]. The gravity simulators at ESA-ESTEC are well accepted standards to prepare for real space flights [25].

We hypothesized, that EC sense and dynamically balance sudden alterations in g-forces and tested, whether short intervals of hyper- or micro-gravity affect endothelial barrier integrity. Our findings were compared to classical mechanical forces to provide a comprehensive picture of the specificity of the endothelial adaptation and capability of ECIS.

## Material and Methods

### Endothelial cell isolation and culture

Human umbilical vein endothelial cells (HUVEC) were isolated from umbilical cords, kindly provided by the Department of Obstetrics, Amstelland Hospital (Amstelveen, The Netherlands) using the method of Jaffe et al. [26] and cultured as described previously [27]. The collection of HUVEC is approved as non-WMO research by the IRB (VU University Medical Center) and written informed consent was provided. HUVEC are continuously collected in our research laboratory, not exclusively for this study and none of the authors had access to any identifying information regarding donors. The primary isolates were grown on standard 6 well culture plates and split in a ratio of 1:3 when reaching confluence ca. five days after initial cell isolation. Passage one was grown to confluence and seeded into the measurement arrays with 60,000 cells/cm^2^. Here the cells were grown for 24 to 48 hours until resistance recordings had shown a stable, confluent endothelial barrier. All surfaces were coated with 1% gelatin. One and a half hours before starting an experiment, growth medium was replaced by hepes buffered experimental medium M199 containing 100 U/mL penicillin, 100 mg/mL streptomycin (all Biowhittaker, Verviers, Belgium) and 1% human serum albumin (HSA, Sanquin, Amsterdam, The Netherlands).

### Electric Cell-substrate Impedance Sensing (ECIS)

ECIS measurements, modeling and analysis were performed as described in detail elsewhere [24]. In general, a single ECIS electrode has a diameter of 250 μm. For hyper-gravity and pressure experiments 8W10E PCB arrays (including 10 electrodes per well), for application of fluid shear stress 1F8x1E PC flow arrays (one electrode) and for simulated micro-gravity special enclosed arrays (10 electrodes per well) [28,29] were used (all Applied BioPhysics, Troy, NY, USA). All measurement arrays were 1% gelatin coated and experiments were started when resistance recordings reached a stable plateau indicating cell confluency. All measurements were performed in single-frequency mode (SFT) at 4000 Hz with maximal temporal resolution for the recording of fast changes, except data for electric modeling, which were acquired in multi-frequency mode (MFT) [24]. Also here sampling time was set to maximum resolution.

### Large Diameter Centrifuge (LDC)

**Fig 1:**
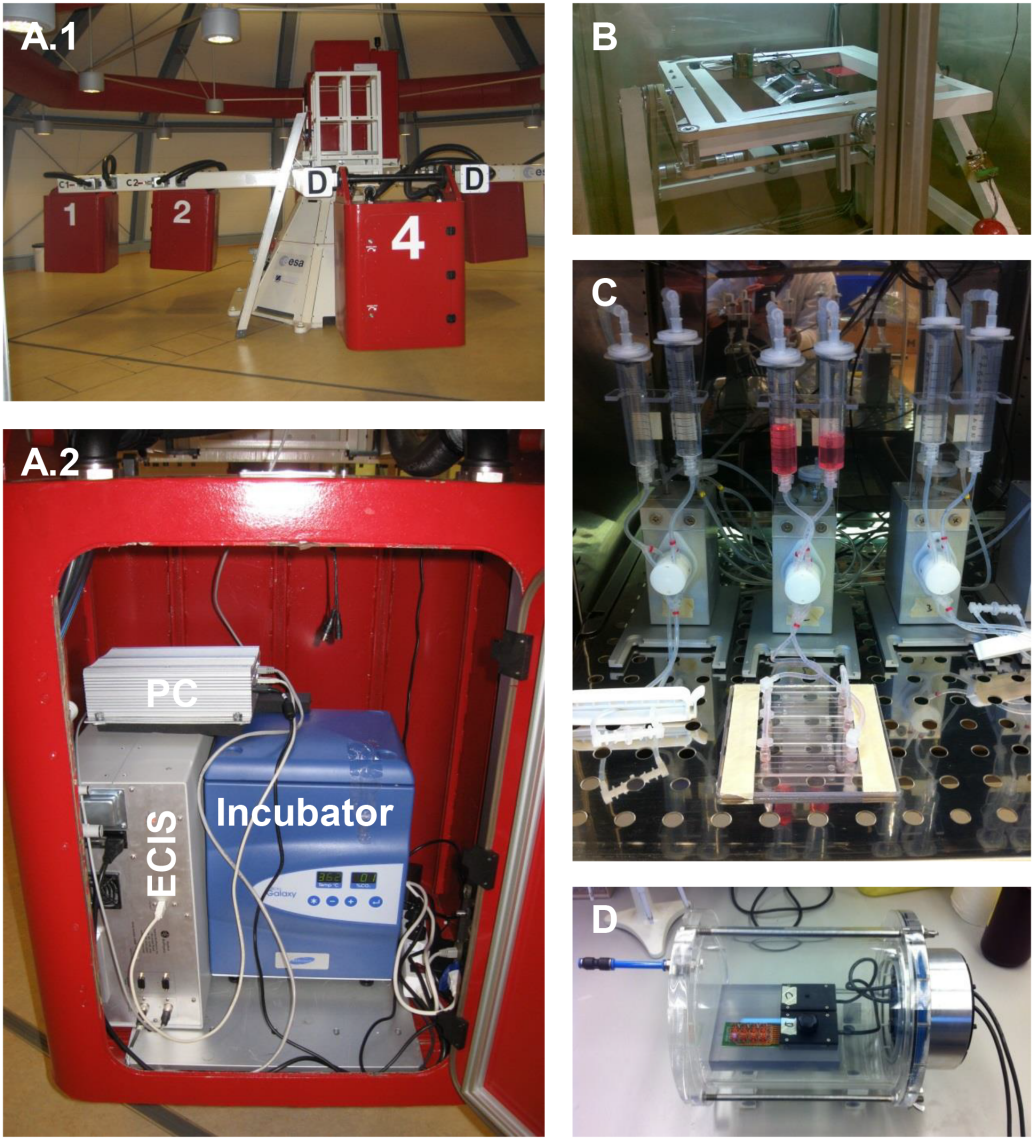
Experimental set-ups. A.1) Large Diameter Centrifuge (LDC) with outer gondolas for application of hyper-gravity and the central gondola for rotational controls. A.2) Measurement set-up for quantification of endothelial characteristics in one of the outer gondolas. The set-up consisted of ECIS system, PC and incubator. B) Random Positioning Machine (RPM) for simulation of near weightlessness. C) Pump-system for application of fluid shear stress. D) Custom made pressure vessel for exertion of hydrostatic pressure.

The LDC at ESA-ESTEC (Noordwijk, The Netherlands) was utilized for the application of hyper-gravity [30]. The LDC with a diameter of 8 meters allows for the application of up to 20g (Fig 1A.1). The four arms of the centrifuge hold at maximum six gondolas, plus one gondola in the center for 1g rotational controls. The gondolas of the centrifuge swing out, resulting in an acceleration vector that is always perpendicular to the samples surface [31]. Dependent on the applied g-forces, the LDC needed about one minutes to reach its final rotational speed. The entire measurement hardware was installed in an outer gondola (Fig 1A.2) and connected to the center for reference measurements at constant 1g (but rotating). Cells were maintained at 37°C without CO_2_ during the experiments.

### Random Positioning Machine (RPM)

The RPM at ESA-ESTEC is a micro-gravity simulator consisting of two independent metal frames that accommodated the sample in their center (Fig 1B) [32]. The metal frames orientate randomly in the three-dimensional space, whereby Earth’s gravity vector is averaged over time. The RPM was operated with a maximal residual acceleration of 0.001g on the sample. The climate chamber of the RPM was set to 37°C without CO_2_.

### Fluid shear stress

The ibidi pump system (Integrated BioDiagnostics, Munich, Germany) was used for the application of laminar fluid shear stress (8 dyn/cm^2^, which equals 0.8 Pa) (Fig 1C). The system is composed of two medium-filled reservoirs, a computer controlled air pump and a special four-fold valve set. By alternating application of air pressure on one of the two reservoirs and a permanent switching of the valves a continuous, uni-directional medium flow was generated over the cells.

### Hydrostatic pressure

A custom made pressure vessel was built from an air-tight acrylic cylinder (Fig 1D) that hosts up to two ECIS arrays and can maintain absolute pressure levels between 0.01 and 5 bar above atmospheric pressure (Mechanical Workshop, VU University Medical Center, Amsterdam, The Netherlands). The HUVEC and medium containing ECIS arrays and array holder were placed inside the pressure vessel and lids were removed. The array holder was connected to the ECIS device through a special pressure tight cable feed through (R125, Roxtec, Harderwijk, The Netherlands). The vessel was closed air tight, placed in a dry air incubator (at 37°C without CO_2_) and connected to the laboratory wall-outlet for compressed air, which provides normal room air with a pressure of 5 bar above atmospheric pressure. The pressure within the vessel was regulated with a Piezo Proportional Pressure Regulator (PR-U08, AirCom Pneumatic GmbH, Ratingen, Germany) and absolute pressures were monitored with an industrial pressure transmitter (DRTR-AL, B+B Thermo-Technik GmbH, Donaueschingen, Germany). Pressure valve and sensor were connected via the NI USB-6008 data acquisition module (National Instruments, Austin, TX, USA) to a computer and controlled with a software written by the Mechanical Workshop in LabVIEW (National Instruments).

### Immunofluorescence staining

HUVEC were cultured on glass slides coated with 1% gelatin and cross-linked with 0.5% glutaraldehyde (Fluka, St. Gallen, Switzerland). The glass slides were handled similar to the ECIS arrays and placed next to the arrays in the LDC. Cells have been fixed with 2% paraformaldehyde in PBS and incubated at room temperature for 30 min. Thereafter fixed cells were washed, membrane permeabilized for one minute with 0.05% Triton X-100 and stained for the junctional protein VE-cadherin (2 μg/mL, 6458, Santa Cruz Biotechnology, Dallas, TX, USA). The F-actin cytoskeleton was visualized with rhodamine-phalloidin (3 U/mL, R415, Life Technologies, Carlsbad, CA, USA) and the nuclei with Hoechst 33258 (0.02 μg/mL, 861405, Sigma-Aldrich). Intensity of the immunofluorescence staining was quantified by manual acquisition of line intensity blots with Slidebook version 5.5 (Intelligent Imaging Innovation, Denver, CO, USA). To this end 5 lines per image were randomly drawn perpendicular to the cell periphery. The measured maximal intensities were averaged to acquire one intensity value per image. This has been repeated for 15 images randomly chosen from 3 donors (5 images per donor).

### Reagents

HUVEC, in the indicated experiments, were pre-incubated for 1 hour with the NO blocker L-NMMA (10 μM, Merck, Billerica, MA, USA) dissolved in experimental medium, before hyper-gravity or fluid shear stress were applied. The L-NMMA supplemented culture medium remained on the cells during the course of the experiments to guarantee stable blockage.

### Statistics

All presented measurements were performed in at least quadruple (replica) on EC from at least three donors (repeats). The exact number of donors is indicated by the number of repeats (n). Resistance was normalized to the average of the presented time interval. Data are represented as mean ± standard error of mean. Statistical differences were calculated by 1-way ANOVA with Bonferroni post-hoc testing or student’s t-test using GraphPad Prism 5 (GraphPad Software, San Diego, CA, USA) and p-values ≤ 0.05 were considered significantly different.

## Results

### Micro- and hyper-gravity cause distinct responses of the endothelial barrier

**Fig 2:**
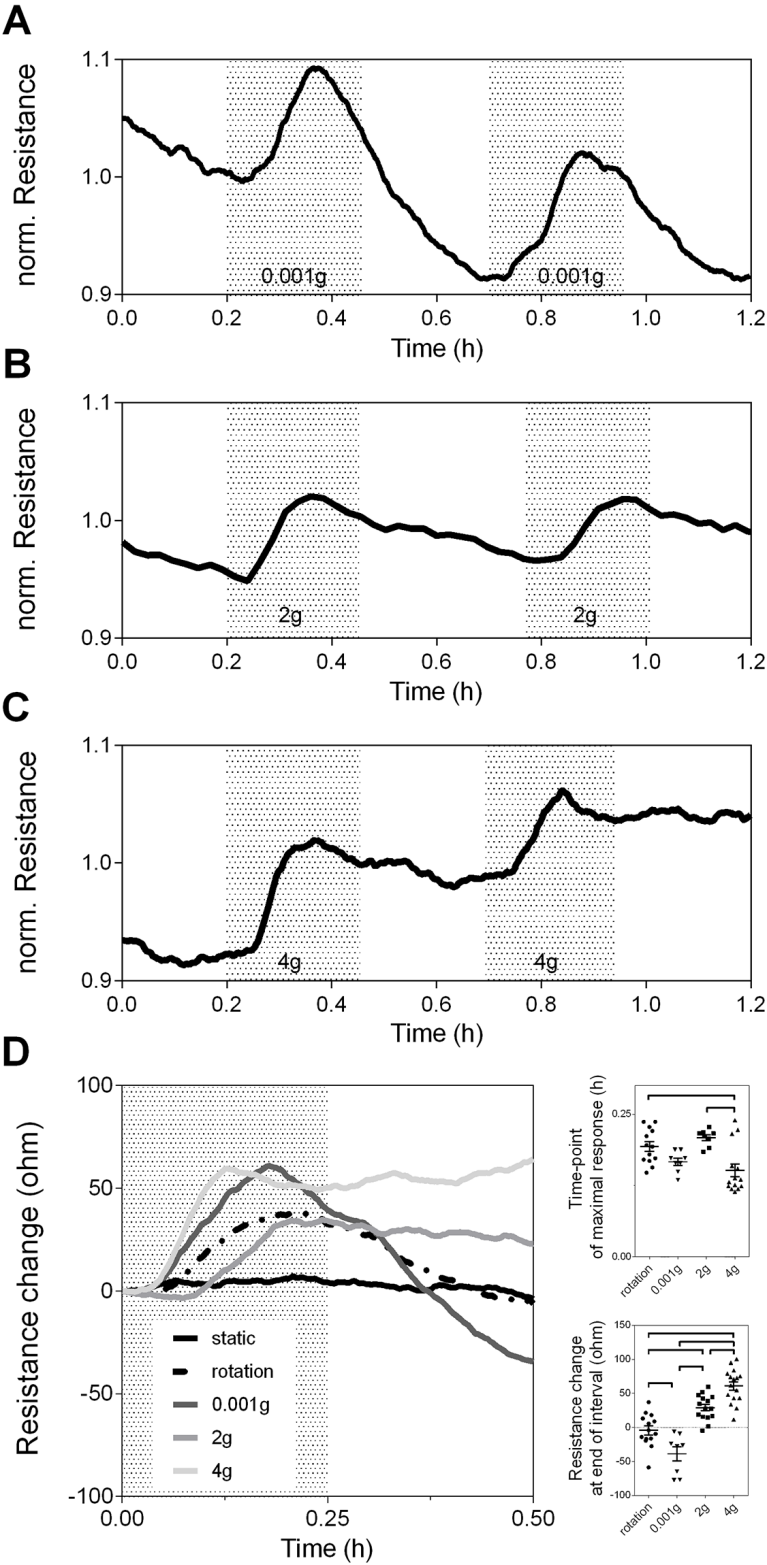
Micro-gravity decreases barrier integrity, whereas hyper-gravity causes sustained and additive improvement of the endothelial barrier. Representative time-course resistance measurements on confluent HUVEC monolayers at alternating intervals of A) 1g and micro-gravity (0.001g), B) 1g and hyper-gravity (2g), or C) 1g and 4g. Gray areas represent intervals of altered g-forces, whereas white areas indicate conditions of 1g. D) Comparison of EC gravity responses vs. static and rotational controls. Data were averaged (n = 4 per group) and presented as change in resistance referred to their baseline value at 0.00 minutes. Graphs to the right show quantifications of time until maximal resistance was reached, and the absolute change in resistance 30 minutes after application of g-forces. Significant differences between the groups are indicated and were calculated by 1-way ANOVA with Bonferroni corrected multiple comparison test.

To test whether alterations in g-forces affect endothelial barrier integrity, confluent HUVEC monolayers were exposed to short intervals (15 minutes) of micro-gravity (0.001g) or hyper-gravity (2 or 4g) (Fig 2). EC responses were evaluated with impedance spectroscopic recordings by defining their time-point of maximal resistance change, time course and resistance values after the application of altered g-forces. The ECIS measurements revealed a steep increase in resistance starting after a lag-time of three to five minutes both following RPM and LDC onset (Fig 2A–2C). The increase in resistance under conditions of micro-gravity reached a maximum after ca. 10 minutes and thereafter started to decrease significantly (p = 0.012 vs. static and rotational control) plateauing at 39.06±10.4 ohms blow baseline values 15 minutes after the RPM was stopped (Fig 2A and 2D). A second interval of micro-gravity induced a similar transient increase in resistance that ultimately remained at the decreased resistance values. In contrast, resistance of EC monolayers under hyper-gravity also responded with an initial transient increase in resistance that started to decline after the resistance maximum was reached, but plateaued at overall increased resistance values 15 minutes after the LDC was stopped (Fig 2B and 2C). Response time and overall resistance increase was found directly related to the magnitude of applied hyper-gravity (Fig 2D). The 4g-condition had a shorter lag-time and reached its resistance maximum significantly faster than the 2g-condition (p = 0.001). In addition, EC exposed to an interval of 2g enhanced their resistance by 29.2±4.8 ohms and 4g by 60.9±6.2 ohms compared to static and rotational controls. The responses to micro- and hyper-gravity were reproducible and repetitive application of 2g and 4g caused additive enhancements of overall barrier integrity.

Surprisingly, the initial, transient raise in resistance was also seen in samples placed in the center of the LDC, where g-forces remained at 1g (but rotating), whereas the static control outside RPM and LDC remained stable and unaffected. Thus, other factors like fluid shear stress or hydrostatic pressure, which could have been induced by rotation of the centrifuge and RPM might affect the endothelial barrier, wherefore we investigated the impact of classical forces on endothelial barrier integrity.

### Shear stress, but not pressure evoke a specific change in electrical barrier integrity

**Fig 3:**
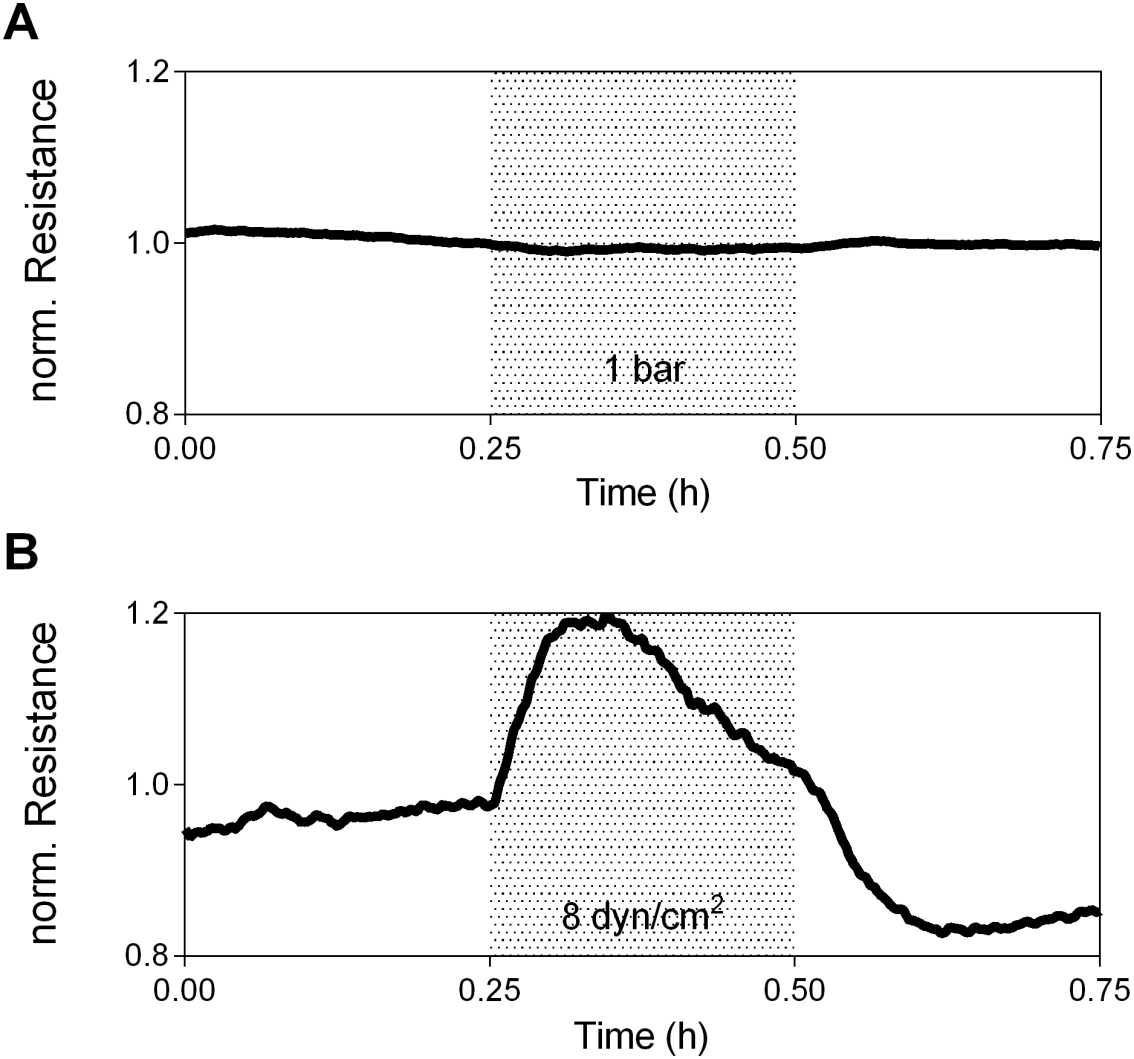
Fluid shear stress induces a specific endothelial response, whereas hydrostatic pressure leaves resistance unaffected. HUVEC were exposed to an interval of A) absolute hydrostatic pressure (1 bar), or B) continuous, uni-directional fluid shear stresses (8 dyn/cm^2^). Gray areas indicate periods of altered forces. Data were averaged (n = 3 per group).

Based on the findings in the rotational controls we tested, whether the effects seen in LDC, RPM and the rotational control can be resembled by classical mechanical forces. Therefore hydrostatic pressure, a force vector perpendicular to area (Fig 3A) was applied to EC in a custom-designed closed vessel and increased to absolute values of 1 bar over normal atmospheric pressure. Resistance did not change in response to increased hydrostatic-pressure excluding the possibility that the observed changes in resistance upon application of g-forces are caused by alterations in pressure.

In addition to pressure, EC are able to sense and precisely adapt to minute changes in shear stress that can arise from the LDC onset [31,33]. Therefore we hypothesized that resistance changes in the LDC might result from inertial shear stresses. Shear stress is a force parallel to area and induced a change in resistance partly resembling the initial response seen under altered conditions of gravity and in the rotational controls (Fig 3B). What sets the shear-response apart, is that resistance increased instantaneously after pump onset, without the previously described lag-time reaching a two-fold higher maximal resistance than in the LDC and RPM, before declining back to values below baseline. As shear stress and pressure did neither cause sustained enhancement nor deterioration of the endothelial barrier as seen in the LDC and RPM, we conclude that the gravity responses are distinct from responses to classical forces.

### Nitric-oxide plays distinct roles in endothelial shear- and gravity-responses

Given the similarities between the initial increase in resistance of the EC barrier after both shear forces and gravitational forces, we hypothesized that similar pathways might get activated in early stages of the adaptive responses.

**Fig 4:**
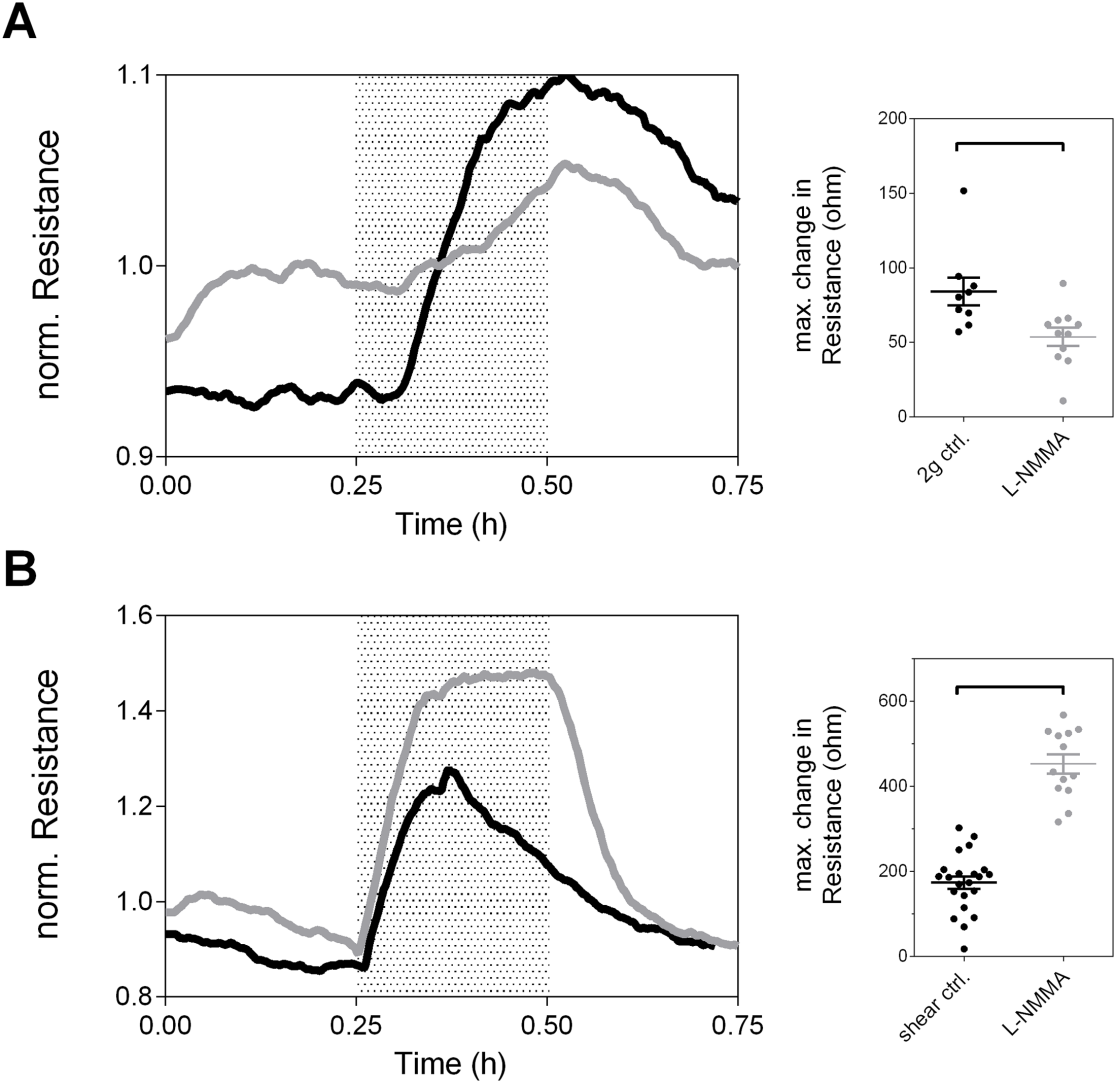
Nitric Oxide does inversely affect endothelial shear- and gravity-responses. Representative ECIS measurements on confluent HUVEC monolayers treated with the nitric oxide blocker L-NMMA (grey curves) compared to controls (black curves) that were subjected to an interval of A) hyper-gravity (2g) or B) fluid hear stress (8 dyn/cm^2^). Dot-plots present quantifications of the absolute, maximal change in resistance with and without the addition of L-NMMA. Statistical differences have been calculated by unpaired student’s t-test.

Nitric-oxide has been indicated as a central mediator of EC barrier- and vaso-regulation in response to mechanical and gravitational stimuli [11,34,3]. Therefore we tested its role in the EC barrier adaptation (Fig 4). Non-treated cultures showed the typical responses to hyper-gravity and shear stress. Inhibition of NO caused a partial blockage of the hyper-gravity response (Fig 4A) resulting in a significantly decreased maximal change in resistance (84.3±9.3 vs. 53.8±6.1 ohms, p = 0.011). In clear contrast, resistance values under shear stress reached ca. 2.5-fold higher maximal resistance values than non-treated controls (173.7±14.7 vs. 452.2±22.5 ohms, p< 0.0001). Importantly, shear-induced resistance values after blocker addition plateaued at their maximal value until the stimulus was switched off and did not show the typical transient behavior. Therefore we concluded that hyper-gravity and shear stress activate distinct signaling pathways in EC.

### Hyper-gravity induces endothelial barrier enhancement mediated by improvement of cell-cell and cell-matrix adhesions

**Fig 5:**
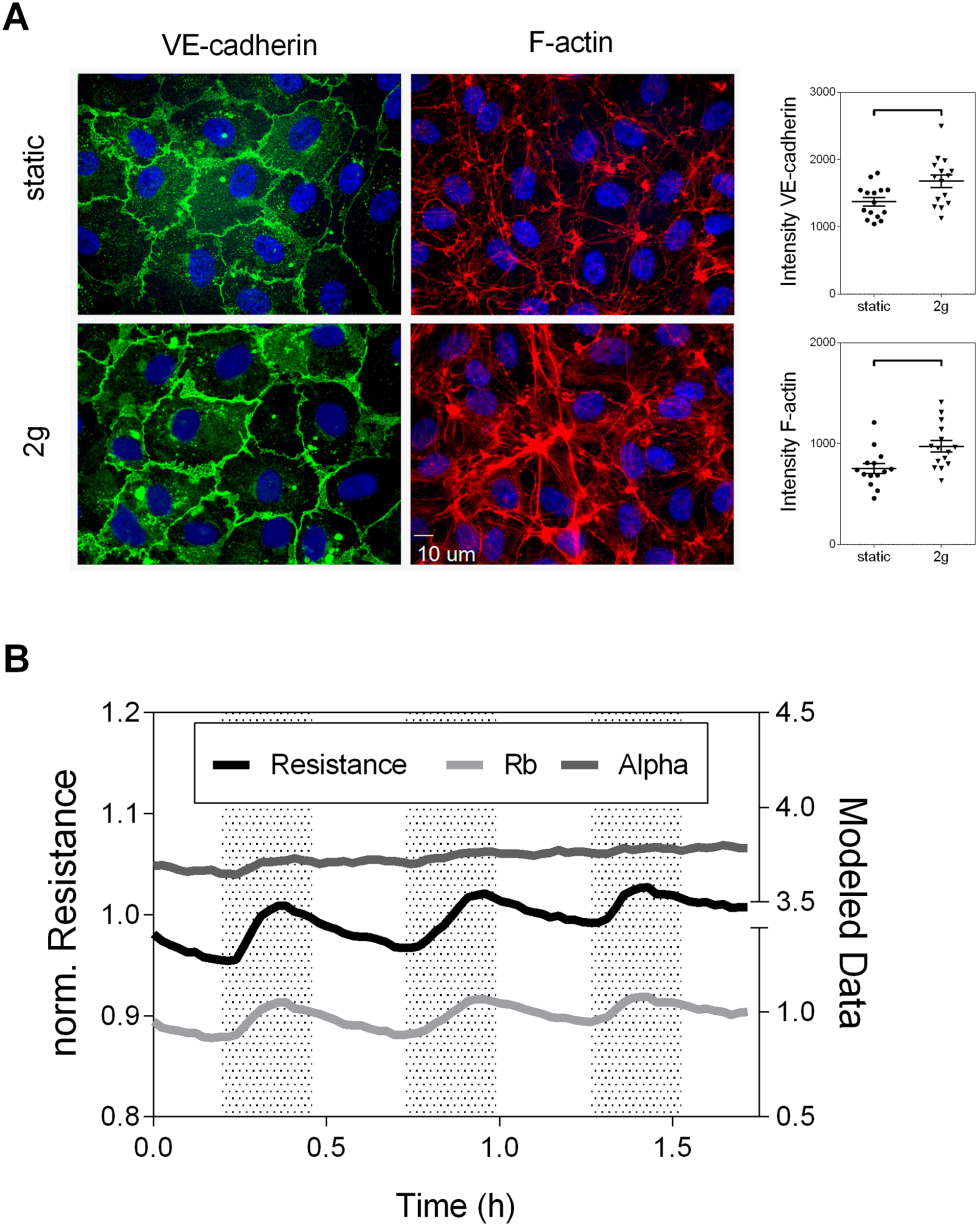
Cell-cell and cell-matrix interactions orchestrate the endothelial gravity-response. A) Immunofluorescence staining of the cell junction protein VE-cadherin and the cytoskeletal protein F-actin, were performed on static controls and after application of hyper-gravity (2g for 15 minutes). The according line intensity quantifications are presented as dot-plots (n = 3 per group). Statistical differences were calculated with unpaired student’s t-test. B) Electrical modeling of ECIS data was carried out to distinguish between the contribution of cell-cell (Rb) and cell-matrix interactions (Alpha) to the overall resistance changes in response to hyper-gravity (2g). Representative data are shown.

The shear-responses did not mimic the EC barrier enhancements under hyper-gravity, wherefore we performed an in-depth analysis of EC behavior in the LDC. To gather insights on the underlying structural changes immunostaining for VE-cadherin and F-actin was performed and quantified (Fig 5A). VE-cadherin, which is involved in the formation and maintenance of EC adherens junctions, showed a significantly enhanced peripheral localization and organization after the application of 2g with parts of the cell membranes overlapping (honeycomb structures) compared to 1g controls that presented with a patchy distribution and lower intensity of the junction protein (1374.0±62.7 vs. 1678.0±93.2, p = 0.011). F-actin content after hyper-gravity was significantly increased and organized in a densely packed peripheral ring, whereas controls showed a normal cytoskeletal arrangement (753.8±50.3 vs. 972.4±57.1, p = 0.008).

Dividing the impedance data in separate components [35,36,24], reflecting cell-cell (Rb) and cell-matrix (Alpha) interactions (Fig 5B), indicated that the characteristic initial, transient raise in resistance is mainly driven by alterations in Rb (para-cellular resistance caused by blockage of current flow through junctional complexes), which rose and fell with the resistance signal. A slower enhancement of cell-matrix adhesions (current flow beneath the cells determined by distance between cells and substrate and focal adhesions) was driving the overall enhancement of the EC barrier in response to hyper-gravity. These findings indicated that the forces applied by the LDC caused an active EC adaptation that is initially driven by fast remodeling of cell-cell adhesions including the accumulation of junctional proteins and accompanied by a slower enhancement of cell-matrix adhesions and enforcement of the peripheral F-actin cytoskeleton.

## Discussion

The main finding of the present study is that EC dynamically adjust their barrier to changes in g-forces. Specifically, short-term application of hyper-gravity caused a rapid, but sustained improvement of endothelial barrier integrity. On the contrary, simulated micro-gravity decreased endothelial resistance and shear stress induced only transient changes in barrier integrity, whereas changes in hydrostatic pressure had no effect. The hyper-gravity response comprised an immediate improvement of cell-cell junctions combined with a slow enhancement of cell-matrix adhesions. Fluid shear stress partly resembled the initial effects of hyper-gravity, however our findings imply that EC gravity- and shear-responses are distinct processes with regard to nitric oxide signaling, whereby our study provides new insights on endothelial gravi-sensing. Furthermore, the here presented experiments are part of a large scale research program that is embedded in the ELIPS ISS biology research program, in which we prepare the ECIS technology for ESA space mission to investigate the role of the endothelium and specifically endothelial junctions in cardiovascular deconditioning under real weightlessness, a major health challenge faced by astronauts. In our study ECIS has shown suitable to characterize subtle gravity-induced adaptations of the endothelial barrier.

Mechanosensing and -transduction, the ability of cells to sense, transmit and adapt to classical mechanical stimuli, is a fundamental function of EC to maintain barrier integrity [37]. Several cellular structures have been shown to play an integrative role in this process, including intercellular junctional complexes, focal adhesions and the cytoskeleton [38]. The vast majority of studies on EC mechanosensing have been carried out under altered conditions of fluid shear stress, whereby gravity-sensors are not defined. Also the initial increase in resistance recorded after the application of hyper-gravity was previously exclusively linked to effects of fluid shear stress. Here, de Paola and colleagues [33] explained, the changes in resistance by mechanical deformation and fast morphological changes of cells leading to a decrease in cell-electrode distance (Alpha) and increased cell radius. Our data show that endothelial cells indeed started to increase their radius (honeycomb structures indicative for overlapping cell peripheries), but merely mechanical deformation does not explain the hyper-gravity induced changes. Specifically the transient nature of the signal, which is independent from the duration of applied force points towards an active, biological adaptation of the EC barrier. Further, we showed that overall resistance improves even after the cells have been returned to normal g-levels, indicating that mere mechanical deformation is not sufficient to explain the barrier changes upon increases in g-forces.

Since mechanical deformation did not explain the barrier improvements, we performed a detailed analysis of the impedance signal that indicated cell-cell adhesions as fast gravity-sensors and -adaptors. VE-cadherin is known for its central role in the maintenance of endothelial barrier integrity by homotypic binding and its mechano-sensory function [39]. Upon application of shear stress VE-cadherin forms a signaling complex with PECAM-1 and VEGFR2 that initiates downstream signaling and shear-adaptation [40]. Our data suggest a role for VE-cadherin under hyper-gravity, as cell-cell interactions (Rb) improved after LDC onset and VE-cadherin accumulated in cell junctions after exposure to hyper-gravity. However, the underlying signaling seems distinct. Further, we found peripheral enforcement of the F-actin cytoskeleton. This observation is supported by a large body of evidence that defines the cytoskeleton as a major responder to gravitational-stimulation and specifically hyper-gravity was shown to increase F-actin content and organization [17,19].

Classically, application of simulated micro-gravity for under 24 hours was shown to causes little effects systemically, whereas detrimental physiological, functional and structural changes were found in humans exposed to long-term weightlessness that were attributed to changed cellular features [41,42]. Our data extend these previous observations and show that short intervals of simulated micro-gravity cause an overall decreased barrier integrity. This is in line with studies showing that early morphological and functional adaptations under micro-gravity cause the activation of mediators like TGF-β1, Caspase-3 and Osteopontin, which reach maximal expression levels at 10 minutes after micro-gravity onset and decline afterwards [43,44]. Therefore release of barrier enhancing mediators could account for the short-term compensation to micro-gravity and the later decrease in overall resistance could be the causative for the reported systemic effects.

Our study bears the limitation that the use of ground-based facilities, to simulate micro- and hyper-gravity provides a close, but incomplete, estimation of the effects seen under real weightlessness [32]. Based on mathematical predictions for the LDC, the shape of our medium reservoirs, the small medium volume and the sample position in the gondola should have prevented development of inertial shear stresses [31]. Likewise the use of special enclosed arrays in the RPM should have prevented fluid movements. Nevertheless, further tests need to exclude the possibility that the initial increase in resistance seen in LDC and RPM are produced by small alterations in fluid shear stress at the onset of rotation (as indicated by the rotational control). Modeling of fluid dynamics will be a helpful tool to work this specific problem out. However, the different effects of NO blockage under shear stress and hyper-gravity argue against shear stress as the source for the experimental changes. We observed NO to be instrumental for the EC gravity-responses, which is supported by findings of increased NO synthesis under hyper-gravity [11,8,18]. On the contrary, blockage of NO showed inverse effects and even enhanced the EC response to shear. Here, the exact signaling mechanisms involved in the distinct effects of NO remain to be fully defined.

## Conclusions

In conclusion, simulated hyper-gravity induces active remodeling of the endothelial barrier, characterized by enhancement of EC junctions, cell-matrix adhesions and cytoskeletal enforcement that resulted in improved barrier integrity, whereas micro-gravity caused decreased endothelial resistance. Further, ECIS has proven capable to continuously monitor subtle EC changes within confluent cell layers under altered conditions of gravity. Therefore the utilization of ECIS at the ISS and in the ground-based facilities at ESA will provide an unique tool to the scientific community to characterize cellular behavior of adherent growing cells under real and simulated hyper- and micro-gravity.
